# Past, present, and future of three‐field lymphadenectomy for thoracic esophageal cancer

**DOI:** 10.1002/ags3.12338

**Published:** 2020-05-14

**Authors:** Harushi Udagawa

**Affiliations:** ^1^ Toranomon Hospital Kajigaya Kawasaki City Japan; ^2^ Okinaka Memorial Institute for Medical Research Tokyo Japan

**Keywords:** recurrent laryngeal nerve chain, supraclavicular fossa, thoracic esophageal cancer, three‐field lymphadenectomy

## Abstract

In spite of repeated appeal of the effectiveness of three‐field lymphadenectomy (3FL) by Japanese esophageal surgeons, it has not been accepted worldwide as a standard therapeutic measure for thoracic esophageal cancer. In this review, a concise summary of the history of 3FL, its present position, and its future perspective is discussed. Although a lack of randomized controlled trial (RCT) is one of the largest criticisms of 3FL, it seems difficult to make 3FL world‐standard even if a RCT with a positive result was made. The essence of 3FL has revealed the fact that bilateral cervical paraesophageal nodes and nodes in the bilateral supraclavicular fossae are regional nodes of thoracic esophageal cancer. To let the world admit this essence should be the real endpoint of “3FL issue” without RCT. In the era of new modalities, Japanese surgeons should be free from the idea that 3FL is indispensable though the essence of 3FL should remain.

## Graphical abstract

A concise summary of the history of 3‐field lymphadenectomy for esophageal cancer, its present position, and its perspective is discussed. Japanese surgeons should be free from the idea that 3FL is indispensable, though the essence of what 3FL has clarified that bilateral cervical paraesophageal nodes and nodes in the bilateral supraclavicular fossae are regional nodes of thoracic esophageal cancer should remain.

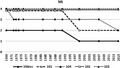

## 1 INTRODUCTION

Although the radical esophagectomy with three‐field lymphadenectomy (3FL) is widely accepted as the standard surgical procedure in the treatment of thoracic esophageal cancer in Japan,^1^ the procedure has long been under criticism^2^, ^3^, ^4^ and is not yet the world‐standard.^5^ However, as experience and the data have accumulated, its position in the therapeutic strategies for treating of esophageal cancer seems to be settled at least in the world of academic literature,^6^, ^7^, ^8^, ^9^, ^10^, ^11^ particularly in Asia. As many reviews and meta‐analyses have been published on this issue, I would like to avoid repetition, and instead share some comments and opinions on the past, present, and future of 3FL from the viewpoint of a Japanese esophageal surgeon.

## 2 PAST

Esophagectomy became one of the routine surgeries for esophageal cancer in 1960s. Following, the 1970s and early 1980s was a time for the pursuit of radicality. With few effective chemotherapeutic agents, surgeons tried to accomplish complete eradication of tumor cells by surgery in many fields. Meticulous and systematic studies on lymph node metastasis revealed the effectiveness of radical lymph node dissection typically in the field of gastric cancer treatment,^12^ and the concept of D2 or D3 lymphadenectomy was established. In the pursuit of radicality of esophageal cancer surgery, the unique position of a lymph node station was recognized. This was a group of nodes located high in the right paratracheal region just behind the root of the right subclavian artery.^13^, ^14^ This position had the highest incidence of lymph node metastasis from thoracic esophageal cancer, and its complete removal seemed to be crucial for better prognosis. This lymph node station, which is now classified as “106recR” by the Japan Esophageal Society^15^ (expressed in this article as “JES classification,” explained in detail in a later paragraph), was given various names by many surgeons such as “highest nodes,” “T(top)‐nodes,” and “106S(superior).” As the investigation proceeded, Japanese surgeons became aware of the close relation of these nodes and the right recurrent laryngeal nerve, and the continuity of these nodes and the cervical paraesophageal nodes (“101” of JES classification^15^) as the recurrent nerve chain.^16^ Because of the bilateral location of the recurrent nerve chain, the left paratracheal region also became included in the dissection, and the prototype of radical superior mediastinal dissection was formed.^17^ In this prototype of radical superior mediastinal dissection, lymph nodes in certain stations (as shown in bold italic font in Table 1) only on the mediastinal side (i.e., excluding “104” and “101” of JES classification) were dissected.

**TABLE 1:** Numbers and descriptions of lymph node stations in JES Classification Numbers and descriptions in bold italic are stations routinely dissected in 3FL. Quoted with modification from 11th edition of Japanese Classification of Esophageal Cancer^15^

	Number of station	Description
Cervical	100	Superficial lymph nodes of the neck
	100spf	Superficial cervical lymph nodes
	100sm	Submandibular lymph nodes
	100tr	Cervical pretracheal lymph nodes
	100ac	Accessory nerve lymph nodes
	***101***	***Cervical paraesophageal lymph nodes***
	***101L***	***Left cervical paraesophageal lymph nodes***
	***101R***	***Right cervical paraesophageal lymph nodes***
	102	Deep cervical lymph nodes
	102up	Upper deep cervical lymph nodes
	102mid	Middle deep cervical lymph nodes
	103	Peripharyngeal lymph nodes
	***104***	***Supraclavicular lymph nodes***
	***104L***	***Left supraclavicular lymph nodes***
	***104R***	***Right supraclavicular lymph nodes***
Superior mediastinal	***105***	***Upper thoracic paraesophageal lymph nodes***
	***106***	***Thoracic paratracheal lymph nodes***
	***106rec***	***Recurrent nerve lymph nodes***
	***106recL***	***Left recurrent nerve lymph nodes***
	***106recR***	***Right recurrent nerve lymph nodes***
	106pre	Pretracheal lymph nodes
	106tb	Tracheobronchial lymph nodes

It had been known that nodes in the supraclavicular fossae or at the bilateral cervical venous angles (“104” of JES Classification^15^) could be involved in the lymphatic spread of the thoracic esophageal cancer,^18^ but had long been considered the sign of far advanced disease and regarded as an expression of systemic tumor spread. The first report of the high incidence of cervical lymph node metastasis from thoracic esophageal cancer and the potential benefit of cervical lymphadenectomy was made by Sannohe.^19^

These two trends were merged and the clinical studies of Japanese style 3FL were started almost simultaneously in many leading hospitals in the early 1980s. Due to the poor recognition of medical statistics, almost all the studies remained phase II trials.^20^, ^21^, ^22^, ^23^ As reports claiming superiority of 3FL compared to the historical control of 2FL accumulated, it became harder and harder for Japanese esophageal surgeons to undergo randomized controlled trial (RCT) between 3FL and 2FL. Detailed data of the distribution of lymph node metastasis were obtained, improved survivals were reported, and operative mortality, although once high, was successfully controlled through many modifications to operative technique and perioperative management.^24^, ^25^

Although we had no evidence from RCTs, we tried to accumulate other types of evidence. In 1991, Isono reported the results of nation‐wide inquiring research, but the results had rather small impact probably because the included interval of the study was too early to get matured results.^26^ Many reports of institutional phase II trials were made.^24^, ^25^ One such report by Kato showed clearly improved prognosis by 3FL in 1996,^27^ but another report by Watanabe in 2000,^28^ analyzing the common database, claimed no difference. This apparent contradiction was understandable to Japanese surgeons because the control groups were not identical, but they raised confusion among foreign readers. These two reports revealed the need to distinguish the effect of complete dissection of bilateral recurrent nerve chain nodes and additional supraclavicular lymph node dissection. However, to differentiate the two was almost impossible because the two were too closely correlated in most of the studies. One small size RCT was reported in 1998 by Nishihira.^29^ In spite of a general trend of better prognosis in the 3FL group, the difference was statistically non‐significant, perhaps because of the small sample size. Since then, many different approaches using clinicopathological data have been reported.^30^, ^31^, ^32^

**FIGURE 1:**
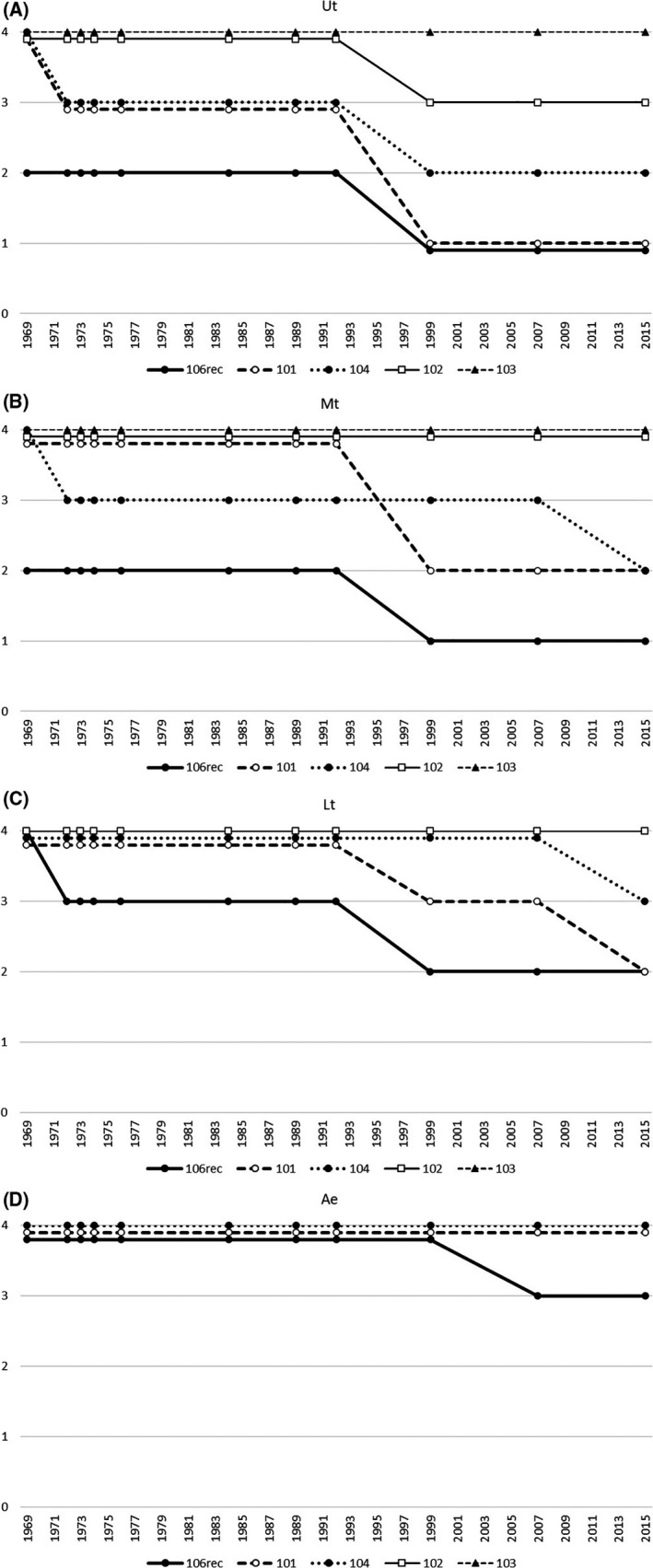
Chronological changes of N‐grading of paratracheal and cervical lymph node stations in JES classification. vertical axis: N‐grade, horizontal axis: year of publication 1st edition: 1969, 2nd: 1972, 3rd :1973, 4th: 1974, 5th: 1976, 6th: 1984, 7th: 1989, 8th: 1992, 9th: 1999, 10th: 2007, 11th: 2015 N3 in the 1st edition is plotted on the level of N4 of later editions. (See text.). (A) Upper thoracic esophageal cancer (Ut). (B) Middle thoracic esophageal cancer (Mt). (C) Lower esophageal cancer (Lt). (D) Abdominal esophageal cancer (Ae)

The Japanese Society for Esophageal Diseases (JSED), which changed its name to the Japan Esophageal Society (JES) in 2003, first issued the common scale for the investigation of esophageal cancer in 1968 and named it “Guide Lines for Clinical and Pathologic Studies on Carcinoma of the Esophagus.” It has been revised many times and is currently in its 11th edition with the name “Japanese Classification of Esophageal Cancer.”^15^, ^33^ These series of editions of esophageal cancer classification are referred to as “JES Classification” or, more simply, “JES‐” as the prefix of a lymph node station in this article. JES classification has changed the categorization of the superior mediastinal and cervical lymph node stations step by step as a new edition was published. Figure 1A‐D shows how such changes have been made. In the 1st edition of the JES classification published in 1969, N‐category was limited from 0 to 3, and, at the time of its publication, N3 meant far advanced disease. Therefore, N3 in the 1st edition is plotted in Figure 1 as if it was N4. It is easily understood that N‐grading number of superior mediastinal and cervical lymph node stations has generally been decreased, with the largest shift occurring in 1999 when the 9th edition was issued. The largest decrease of N‐number occurred in JES‐“101” (cervical paraesophageal nodes) with the recognition of the importance of recurrent laryngeal nerve chain. This cervical paraesophageal node station was clearly included in the regional nodes of thoracic esophageal cancer in the 7th UICC‐TNM system in 2009,^34^ but supraclavicular node group (JES‐“104”) is still classified as extra‐regional nodes in the latest 8th edition regardless of the location of the tumor in the thorax in spite of much argument by Japanese surgeons.^30^, ^31^, ^32^, ^35^

Japanese surgeons’ preference of radical surgery represented by 3FL was once challenged by Japanese medical oncologists and radiologists in the 2000s. In 2003, Hironaka and Ohtsu reported^36^ a relatively good survival result of their small phase II study of definitive chemoradiation therapy, and claimed that the result had no statistically significant difference from that of their historical control of surgically treated patients. This report made a large temporary shift of patients from radical surgery to definitive chemoradiotherapy. In order to confirm this result, a larger scale phase II trial of JCOG9906 was carried out. The result of JCOG9906^37^ was worse than that of the pilot study by Hironaka. At almost the same time, Ando reported the result of JCOG9907,^38^ which compared survivals between neoadjuvant chemotherapy followed by radical surgery and radical surgery followed by adjuvant chemotherapy. Because the inclusion criteria of JCOG9906 and 9907 were very similar, the results of these two studies gathered large attention. The result was a preference for the test arm of JCOG9907, or neoadjuvant chemotherapy followed by surgery, which showed a 55% (46.7–62.5%) 5‐year overall survival rate, which was apparently better than the 36.8% (26.1–47.5%) of JCOG9906. Although these figures cannot be compared directly because the studies were separate, these results influenced another shift of patients back to surgery. Many reports on the increased risk of salvage surgery after definitive chemoradiation^39^, ^40^ acted as another wind for the surgery‐oriented strategy.

## 3 PRESENT

Many meta‐analyses and recent studies comparing 3FL and 2FL^6^, ^7^, ^8^, ^9^, ^10^, ^11^ generally report the tendency of better prognosis of the 3FL group. However, most of them have been written by Asian investigators, though some reports from Western countries also support the superiority of 3FL.^41^, ^42^ According to the analyses of our substantial data,^32^ cervical paraesophageal nodes (JES‐“101”) have non‐negligible Efficacy Index for upper, middle, and even lower esophageal cancer. But the Efficacy Index of nodes in supraclavicular fossae (JES‐“104”) for lower esophageal cancer is negligibly low. Tachimori reported the result of a similar study in 2016,^31^ which used the larger, nation‐wide database of JES registration. Although the approach of the two studies was similar, the calculated figures of the Efficacy Index by Tachimori were much higher. A possible explanation for this is that 3FL had been routinely applied to patients in Udagawa's report, while the data of JES registration in Tachimori's report had been much more biased by the hidden indication of 3FL in many different institutions, such as “applied only when cervical lymph node metastasis had been suspected preoperatively.” Both reports concluded that JES‐“104” should be included in the regional lymph nodes of the thoracic esophagus, and that 3FL is strongly recommended for esophageal cancer located in the upper thoracic esophagus, is feasible for tumor in mid‐thoracic esophagus, but its feasibility is not clear for tumor in the lower thoracic esophagus.^30^, ^31^, ^32^

There was another relatively large change in JES classification when its 11th edition was published in 2015. In the 11th edition, JES‐“104” was defined as N2 for tumors in the middle thoracic esophagus and N3 for tumors in the lower esophagus, and JES‐“101” became N2 for tumors in the lower esophagus. This means that bilateral JES‐“101” nodes should be dissected no matter if it is done from the mediastinal side or cervical side to accomplish D2 dissection for thoracic esophageal cancer including lower esophageal cancer, and 3FL is mandatory for middle thoracic esophageal cancer to make your dissection D2.

As discussed above, JES‐“101” (cervical paraesophageal nodes) and JES‐“104” (nodes in supraclavicular fossae) should be discussed separately. With the advance of operative technique particularly after the introduction of video‐assisted thoracic approach, surgeons began to feel that they could dissect nodes along the bilateral recurrent laryngeal nerves up to a much higher level than before. In the past age of open surgery, the nodes along the bilateral recurrent laryngeal nerves were JES‐“106rec” when dissected from the thoracic side and JES‐“101” when dissected from the cervical side. Actually, the recurrent nerve chain nodes are continual and there is no clear border line recognizable during operation between JES‐ “106rec” and JES‐“101.” These days, many surgeons think that these nodes on the right side can all be dissected completely from the thoracic side with video‐assisted technique or even with open thoracotomy.^43^ Some surgeons claim that they can clear up also the left recurrent nerve chain nodes with thoracoscopic or robotic approach.^44^ If this is true, the mostly updated 2FL can include all JES‐“101” nodes in its dissection field, and converting it to 3FL only means the mere addition of bilateral JES‐“104”. This means that we have to re‐evaluate the value of 3FL compared to 2FL. Adding to this, many reports have been trying to reduce the indication of 3FL by examining paratracheal lymph nodes dissected in the thoracic phase of the operation, regarding them as an indicator of possible tumor involvement in the supraclavicular nodes,^45^, ^46^, ^47^ although a warning against this strategy has been made from the result of sentinel node study.^48^ Many reports have been made which re‐evaluate and deny or limit the value of 3FL.^49^, ^50^, ^51^, ^52^ Some claim that because of the unique position of cervical (JES‐“104”) lymph nodes, their metastases are easily detected by routine examination such as ultrasonography and the additional dissection of the supraclavicular fossa after the suspicion of metastasis rises is usually not difficult.^53^ There are several reports that cervical dissection deteriorates the swallowing function.^54^, ^55^ I personally think the main cause of the deterioration is the wide and thorough dissection of bilateral recurrent nerve chains, and that the addition of bilateral supraclavicular fossa dissection does not play an important role in this phenomenon. As is well known, the ventral‐dorsal positioning of nodes along the recurrent laryngeal nerves are different according to laterality.^56^, ^57^ Nodes on the left side of the trachea are more frequently located anterior to the left recurrent laryngeal nerve and this phenomenon becomes more obvious as the nodes are located in higher position. Although JES‐“101L” is described as left cervical paraesophageal nodes, its essential pathophysiological definition should be “the left recurrent nerve chain nodes in the cervical region”. It is located usually anterior to the left recurrent laryngeal nerve and thus just on the left lateral or even anterior wall of the trachea. It is somewhat confusing because there is another lymph node station (JES‐“100tr”) described as cervical pretracheal nodes. It is important to know that such left cervical recurrent nerve chain nodes are usually classified as JES‐“101L,” not as JES‐“100tr.” Therefore, I think it is necessary to approach from the cervical side to remove JES‐“101” nodes completely on the left side. Even on the right side, the highest node located just on the dorsal side of the recurrent nerve entering the larynx seems difficult to dissect from the thoracic side and it can be much safer to approach from the cervical side to avoid recurrent nerve palsy. Therefore, we still make it routine at our institution to apply 3FL to advanced thoracic esophageal cancer. However, as it is also true that a large portion of JES‐“101” can be dissected from the thoracic side, particularly on the right side, and as the metastases are dominant on the right, I suspect that a very large number of patients is needed to show the statistically significant survival benefit of 3FL if it is compared to 2FL with meticulous superior mediastinal dissection including most but not all of JES‐“101.”

As discussed above, there are many factors within this issue that require our attention. We know that one very interesting RCT between 3FL and 2FL is underway in China,^58^ but its result, no matter positive or negative, should be examined carefully and critically.

## 4 FUTURE

Even if the most recent Chinese RCT^58^ results in the superiority of 3FL, because of dominance of adenocarcinoma in Western countries and their relatively low localization, it seems very difficult for surgeons in Western countries to accept 3FL as a standard surgery. On the other hand, squamous cell carcinoma of the esophagus seems to be regarded more as a disease for chemoradiotherapy than surgery, particularly in the United States. However, just as is reflected in the definition of regional nodes of the TNM system, at least the importance of superior mediastinal lymph nodes, including cervical paraesophageal nodes, has been accepted by Western specialists of esophageal cancer; as a next step, Japanese surgeons hope they at least include nodes in the supraclavicular fossae in the category of regional nodes of upper to middle thoracic esophageal cancer. For this reason, our participation in international studies such as “Worldwide Esophageal Cancer Collaboration,”^59^ “International Esodata Study,”^60^ and “TIGER study”^61^ is very important. While we are strangers, what we talk about may sound like a myth, but after we know each other, data will talk. I think it should be the real endpoint of “3FL issue” without RCT.

At the same time, advancement of diagnostic measures is very important. Until now, almost all the studies on diagnostic measures have revealed such measures to be far from sufficient to utilize them for the selection of suitable patients for 3FL. Only precise histopathological depth diagnosis of a very early tumor made by endoscopic removal such as EMR (endoscopic mucosal resection) or ESD (endoscopic submucosal dissection) can properly predict the possibility of metastasis.^62^ Sentinel node concept seems to be useful in selecting candidates for less invasive treatment from patients with relatively early stage tumors.^48^, ^63^, ^64^ However, these strategies utilizing EMR, ESD or sentinel node concept can all be feasible in early‐stage diseases and not many patients can benefit from them. If we can find an accurate diagnostic strategy to select patients who really need 3FL, 3FL will be more easily accepted.

Japanese surgeons should more deeply consider the implication of 3FL. Even after JES‐“101” and JES‐“104” are admitted as regional nodes, the therapeutic approach can be various including surgery, radiation, chemotherapy, and other newer modalities. The analysis of data obtained by 3FL tells us that JES‐“101” and JES‐“104” are regional nodes. Surgical removal seems more reliable than other measures such as chemotherapy or radiotherapy. However, all such facts are stated in terms of relativity, and all the therapeutic measures are associated with complications. If some therapy other than surgery can yield similar or better results with less complications, it can replace the role of 3FL. The current standard treatment strategy for thoracic esophageal cancer in Japan (i.e., neoadjuvant chemotherapy with 5FU and cisplatin followed by radical surgery represented by 3FL) is questioned by Western standards, and the result of JCOG1109^65^ is awaited. We have to focus on the pathological results of JES‐“101” and JES‐“104” of the protocol with best survival rate proved in JCOG1109 and compare them to their counterparts of the JCOG9907. This investigation may further change the value of 3FL. At the same time, we have to evaluate many other newer treatments and their combination with current therapeutic strategies including definitive chemoradiation because there have also been many advances in the field of chemoradiation. Now, we have high confidence in our surgery, in its effectiveness and safety. But if we stick to it too adamantly, it looks like a kind of religion and too far from science. It is true that the majority of Japanese surgeons expect that 3FL will remain in the mainstream of surgical options, but I honestly think that the possibility may not be so high. Instead, the essence of 3FL – that cervical paraesophageal nodes and nodes in the supraclavicular fossae are the regional nodes of thoracic esophageal cancer – should remain.

## CONFLICT OF INTEREST

The author declares no conflict of interests for this article.

## References

[1] Kitagawa Y , Uno T , Oyama T , Kato K , Kato H , Kawakubo H , et al. Esophageal cancer practice guidelines 2017 edited by the Japan esophageal society: part 2. Esophagus. 2019;16(1):25–43. 30171414 10.1007/s10388-018-0642-8 PMC6510875

[2] Rice TW . Superficial oesophageal carcinoma: is there a need for three‐field lymphadenectomy? Lancet. 1999;354(9181):792–4. 10485718 10.1016/S0140-6736(99)80005-1

[3] Law S , Wong J . Current management of esophageal cancer. J Gastrointest Surg. 2005;9(2):291–310. 15694827 10.1016/j.gassur.2004.06.007

[4] Cense HA , van Eijck CH , Tilanus HW . New insights in the lymphatic spread of oesophageal cancer and its implications for the extent of surgical resection. Best Pract Res Clin Gastroenterol. 2006;20(5):893–906. 16997168 10.1016/j.bpg.2006.03.010

[5] Boone J , Livestro DP , Elias SG , Borel Rinkes IH , van Hillegersberg R . International survey on esophageal cancer: part I surgical techniques. Dis Esophagus. 2009;22(3):195–202. 19191856 10.1111/j.1442-2050.2008.00929.x

[6] Ye T , Sun Y , Zhang Y , Zhang Y , Chen H . Three‐field or two‐field resection for thoracic esophageal cancer: a meta‐analysis. Ann Thorac Surg. 2013;96(6):1933–41. 24055234 10.1016/j.athoracsur.2013.06.050

[7] Ma GW , Situ DR , Ma QL , Long H , Zhang LJ , Lin P , et al. Three‐field vs two‐field lymph node dissection for esophageal cancer: a meta‐analysis. World J Gastroenterol. 2014;20(47):18022–30. 25548502 10.3748/wjg.v20.i47.18022 PMC4273154

[8] Saravanan MN . Two field versus three field lymphadenectomy in carcinoma esophagus: current perspectives. Clinic Surg. 2016;1:1–3.

[9] Shang QX , Chen LQ , Hu WP , Deng HY , Yuan Y , Cai J . Three‐field lymph node dissection in treating the esophageal cancer. J Thorac Dis. 2016;8(10):E1136–E1149. 27867579 10.21037/jtd.2016.10.20 PMC5107465

[10] Matsuda S , Takeuchi H , Kawakubo H , Kitagawa Y . Three‐field lymph node dissection in esophageal cancer surgery. J Thorac Dis. 2017;9(Suppl 8):S731–S740. 28815069 10.21037/jtd.2017.03.171 PMC5538994

[11] Shao L , Ye T , Ma L , Lin D , Hu H , Sun Y , et al. Three‐field versus two‐field lymph node dissection for thoracic esophageal squamous cell carcinoma: a propensity score‐matched comparison. J Thorac Dis. 2018;10(5):2924–32. 29997958 10.21037/jtd.2018.05.69 PMC6006065

[12] Okajima K . Surgical treatment of gastric cancer with special reference to lymph node removal. Acta Med Okayama. 1977;31(6):369–82. 147606

[13] Kinoshita I , Ohashi I , Nakagawa K , Kajitani T . Lymph node metastasis in esophageal cancer; with special reference to upper mediastinum and measures for its treatment (Jpn.). The Japanese Journal of Gastroenterological. Surgery. 1976;9(4):424–30.

[14] Nishihira T , Sayama J , Ueda H . Lymph flow and lymph node metastasis in esophageal cancer. Surg Today. 1995;25(4):307–17. 7633121 10.1007/BF00311252

[15] Japan Esophageal Society . Japanese Classification of Esophageal Cancer, 11th Edition: part I. Esophagus. 2017;14(1):1–36. 28111535 10.1007/s10388-016-0551-7 PMC5222932

[16] Haagensen CD , Feind CR , Herter FP . The lymphatics in cancer. Philadelphia, PA: WB Saunders Co; 1972.

[17] Akiyama H . Surgery for carcinoma of the esophagus. Curr Probl Surg. 1980;17(2):53–120. 6987040 10.1016/s0011-3840(80)80025-6

[18] Ide H , Endo M , Hanyu F , Sakakibara N , Kinoshita Y , Suzuki H , et al. Lymph node metastasis of thoracic esophageal cancer (JPN). Operation (Shujutsu). 1974;28(12):1355–64.

[19] Sannohe Y , Hiratsuka R , Doki K . Lymph node metastases in cancer of the thoracic esophagus. Am J Surg. 1981;141(2):216–8. 7457740 10.1016/0002-9610(81)90160-4

[20] Ando N , Shinozawa Y , Kikunaga H , Koyama Y , Nagashima A , Osaku M , et al. An assessment of extended lymphadenectomy including cervical node dissection for cancer of the thoracic esophagus. Nihon Geka Gakkai Zasshi. 1989;90(9):1616–8. 2586479

[21] Fujita H , Kakegawa T , Yamana H , Shirouzu G , Minami T , Ono T , et al. Cervico‐thoracic‐abdominal lymph node dissection for carcinoma of the thoracic esophagus. Nihon Geka Gakkai Zasshi. 1989;90(9):1623–5. 2586481

[22] Makuuchi H , Machimura T , Sugihara T , So Y , Sasaki T , Tajima T , et al. advantages and disadvantages of three regional lymph node dissection of thoracic esophageal carcinoma and the lymph node dissection by thoraco‐abdomino‐midsternal approach. Nihon Geka Gakkai Zasshi. 1989;90(9):1630–4. 2586483

[23] Akiyama H . Surgery for cancer of the Esophagus, 1990;124–6.

[24] Fujita H , Sueyoshi S , Tanaka T , Shirouzu K . Three‐field dissection for squamous cell carcinoma in the thoracic esophagus. Ann Thorac Cardiovasc Surg. 2002;8(6):328–35. 12517291

[25] Udagawa H , Akiyama H . Surgical treatment of esophageal cancer: Tokyo experience of the three‐field technique. Dis Esophagus. 2001;14(2):110–4. 11553219 10.1046/j.1442-2050.2001.00166.x

[26] Isono K , Sato H , Nakayama K . Results of a nationwide study on the three‐field lymph node dissection of esophageal cancer. Oncology. 1991;48(5):411–20. 1745490 10.1159/000226971

[27] Kato H , Tachimori Y , Watanabe H , Igaki H , Nakanishi Y , Ochiai A . Recurrent esophageal carcinoma after esophagectomy with three‐field lymph node dissection. J Surg Oncol. 1996;61(4):267–72. 8627996 10.1002/(SICI)1096-9098(199604)61:4<267::AID-JSO6>3.0.CO;2-8

[28] Watanabe H , Kato H , Tachimori Y . Significance of extended systemic lymph node dissection for thoracic esophageal carcinoma in Japan. Recent Results Cancer Res. 2000;155:123–33. 10693246 10.1007/978-3-642-59600-1_13

[29] Nishihira T , Hirayama K , Mori S . A prospective randomized trial of extended cervical and superior mediastinal lymphadenectomy for carcinoma of the thoracic esophagus. Am J Surg. 1998;175(1):47–51. 9445239 10.1016/s0002-9610(97)00227-4

[30] Tachimori Y . Pattern of lymph node metastases of squamous cell esophageal cancer based on the anatomical lymphatic drainage system: efficacy of lymph node dissection according to tumor location. J Thorac Dis. 2017;9(Suppl 8):S724–S730. 28815068 10.21037/jtd.2017.06.19 PMC5538982

[31] Tachimori Y , Ozawa S , Numasaki H , Matsubara H , Shinoda M , Toh Y , et al. Efficacy of lymph node dissection by node zones according to tumor location for esophageal squamous cell carcinoma. Esophagus. 2016;13:1–7. 26752982 10.1007/s10388-015-0515-3 PMC4698372

[32] Udagawa H , Ueno M , Shinohara H , Haruta S , Kaida S , Nakagawa M , et al. The importance of grouping of lymph node stations and rationale of three‐field lymphoadenectomy for thoracic esophageal cancer. J Surg Oncol. 2012;106(6):742–7. 22504922 10.1002/jso.23122

[33] Japan Esophageal Society . Japanese classification of esophageal cancer, 11th Edition: part II and III. Esophagus. 2017;14(1):37–65. 28111536 10.1007/s10388-016-0556-2 PMC5222925

[34] International Union Against Cancer . Oesophagus including oesophagogastric junction In: Sobin LH , Gospodarowicz MK , Wittekind C , editors. TNM classification of malignant tumours, 7th edn West Sussex, UK: Wiley‐Blackwell; 2009 p. 66–72.

[35] Udagawa H , Ueno M . Comparison of two major staging systems of esophageal cancer‐toward more practical common scale for tumor staging. Ann Transl Med. 2018;6(4):76. 29666799 10.21037/atm.2018.01.27 PMC5890035

[36] Hironaka S , Ohtsu A , Boku N , Muto M , Nagashima F , Saito H , et al. Nonrandomized comparison between definitive chemoradiotherapy and radical surgery in patients with T(2–3)N(any) M(0) squamous cell carcinoma of the esophagus. Int J Radiat Oncol Biol Phys. 2003;57(2):425–33. 12957254 10.1016/s0360-3016(03)00585-6

[37] Kato K , Muro K , Minashi K , Ohtsu A , Ishikura S , Boku N , et al. Phase II study of chemoradiotherapy with 5‐fluorouracil and cisplatin for Stage II‐III esophageal squamous cell carcinoma: JCOG trial (JCOG 9906). Int J Radiat Oncol Biol Phys. 2011;81(3):684–90. 20932658 10.1016/j.ijrobp.2010.06.033

[38] Ando N , Kato H , Igaki H , Shinoda M , Ozawa S , Shimizu H , et al. A randomized trial comparing postoperative adjuvant chemotherapy with cisplatin and 5‐fluorouracil versus preoperative chemotherapy for localized advanced squamous cell carcinoma of the thoracic esophagus (JCOG9907). Ann Surg Oncol. 2012;19(1):68–74. 21879261 10.1245/s10434-011-2049-9

[39] Swisher SG , Marks J , Rice D . Salvage esophagectomy for persistent or recurrent disease after definitive chemoradiation. Ann Cardiothorac Surg. 2017;6(2):144–51. 28447003 10.21037/acs.2017.03.02 PMC5387142

[40] Nishimura M , Daiko H , Yoshida J , Nagai K . Salvage esophagectomy following definitive chemoradiotherapy. Gen Thorac Cardiovasc Surg. 2007;55(11):461–4. 18049854 10.1007/s11748-007-0157-z

[41] Altorki N , Kent M , Ferrara C , Port J . Three‐field lymph node dissection for squamous cell and adenocarcinoma of the esophagus. Ann Surg. 2002;236(2):177–83. 12170022 10.1097/00000658-200208000-00005 PMC1422563

[42] Lerut T , Nafteux P , Moons J , Coosemans W , Decker G , De Leyn P , et al. Three‐field lymphadenectomy for carcinoma of the esophagus and gastroesophageal junction in 174 R0 resections: impact on staging, disease‐free survival, and outcome: a plea for adaptation of TNM classification in upper‐half esophageal carcinoma. Ann Surg. 2004;240(6):962–72. 15570202 10.1097/01.sla.0000145925.70409.d7 PMC1356512

[43] Yagi D , Hosogi H , Akagawa S , Kawada H , Shimoike N , Kanaya S . Is complete right cervical paraesophageal lymph node dissection possible in the prone position during thoracoscopic esophagectomy? Esophagus. 2019;16(3):324–9. 30945097 10.1007/s10388-019-00664-1

[44] Chao YK , Hsieh MJ , Liu YH , Liu HP . Lymph Node Evaluation in Robot‐Assisted Versus Video‐Assisted Thoracoscopic Esophagectomy for Esophageal Squamous Cell Carcinoma: A Propensity‐Matched Analysis. World J Surg. 2018;42(2):590–8. 28801820 10.1007/s00268-017-4179-0

[45] Shiozaki H , Yano M , Tsujinaka T , Inoue M , Tamura S , Doki Y , et al. Lymph node metastasis along the recurrent nerve chain is an indication for cervical lymph node dissection in thoracic esophageal cancer. Dis Esophagus. 2001;14(3–4):191–6. 11869318 10.1046/j.1442-2050.2001.00206.x

[46] Sato F , Shimada Y , Li Z , Kano M , Watanabe G , Maeda M , et al. Paratracheal lymph node metastasis is associated with cervical lymph node metastasis in patients with thoracic esophageal squamous cell carcinoma. Ann Surg Oncol. 2002;9(1):65–70. 11829432 10.1245/aso.2002.9.1.65

[47] Xu J , Zheng B , Zhang S , Zeng T , Chen H , Zheng W , et al. The clinical significance of the intraoperative pathological examination of bilateral recurrent laryngeal nerve lymph nodes using frozen sections in cervical field lymph node dissection of thoracic esophageal squamous cell carcinoma. J Thorac Dis. 2019;11(8):3525–33. 31559059 10.21037/jtd.2019.07.59 PMC6753458

[48] Takeuchi H , Fujii H , Ando N , Ozawa S , Saikawa Y , Suda K , et al. Validation study of radio‐guided sentinel lymph node navigation in esophageal cancer. Ann Surg. 2009;249(5):757–63. 19387329 10.1097/SLA.0b013e3181a38e89

[49] Law S , Wong J . Two‐field dissection is enough for esophageal cancer. Dis Esophagus. 2001;14(2):98–103. 11553217 10.1046/j.1442-2050.2001.00164.x

[50] Nozoe T , Kakeji Y , Baba H , Maehara Y . Two‐field lymph‐node dissection may be enough to treat patients with submucosal squamous cell carcinoma of the thoracic esophagus. Dis Esophagus. 2005;18(4):226–9. 16128778 10.1111/j.1442-2050.2005.00482.x

[51] Fang WT , Chen WH , Chen Y , Jiang Y . Selective three‐field lymphadenectomy for thoracic esophageal squamous carcinoma. Dis Esophagus. 2007;20(3):206–11. 17509116 10.1111/j.1442-2050.2007.00671.x

[52] Shim YM , Kim HK , Kim K . Comparison of survival and recurrence pattern between two‐field and three‐field lymph node dissections for upper thoracic esophageal squamous cell carcinoma. J Thorac Oncol. 2010;5(5):707–12. 20421764 10.1097/JTO.0b013e3181d3ccb2

[53] Koterazawa Y , Oshikiri T , Takiguchi G , Hasegawa H , Yamamoto M , Kanaji S , et al. Prophylactic cervical lymph node dissection in thoracoscopic esophagectomy for esophageal cancer increases postoperative complications and does not improve survival. Ann Surg Oncol. 2019;26(9):2899–904. 31187365 10.1245/s10434-019-07499-1

[54] Yasuda T , Yano M , Miyata H , Yamasaki M , Takiguchi S , Fujiwara Y , et al. Evaluation of dysphagia and diminished airway protection after three‐field esophagectomy and a remedy. World J Surg. 2013;37(2):416–23. 23052815 10.1007/s00268-012-1822-7

[55] Kumai Y , Samejima Y , Watanabe M , Yumoto E . Videofluoroscopic evaluation of pharyngeal swallowing dysfunction after esophagectomy with three‐field lymph node dissection. Eur Arch Otorhinolaryngol. 2017;274(1):321–6. 27423640 10.1007/s00405-016-4209-9

[56] Ninomiya I , Okamoto K , Fujimura T , Fushida S , Osugi H , Ohta T . Oncologic outcomes of thoracoscopic esophagectomy with extended lymph node dissection: 10‐year experience from a single center. World J Surg. 2014;38(1):120–30. 24101019 10.1007/s00268-013-2238-8

[57] Kanemura T , Makino T , Miyazaki Y , Takahashi T , Kurokawa Y , Yamasaki M , et al. Distribution patterns of metastases in recurrent laryngeal nerve lymph nodes in patients with squamous cell esophageal cancer. Dis Esophagus. 2017;30(1):1–7. 10.1111/dote.12527 27630087

[58] Chen H .Esophagectomy: three‐field Versus Two‐field Lymphadenectomy (NCT01807936, ClinicalTrials.gov in the National Library of Medicine). https://clinicaltrials.gov/ct2/show/NCT01807936

[59] Rice TW , Rusch VW , Apperson‐Hansen C , Allen MS , Chen LQ , Hunter JG , et al. Worldwide esophageal cancer collaboration. Dis Esophagus. 2009;22(1):1–8. 19196264 10.1111/j.1442-2050.2008.00901.x

[60] Low DE , Kuppusamy MK , Alderson D , Cecconello I , Chang AC , Darling G , et al. Benchmarking Complications Associated with Esophagectomy. Ann Surg. 2019;269(2):291–8. 29206677 10.1097/SLA.0000000000002611

[61] Hagens ERC , van Berge Henegouwen M , van Sandick JW , Cuesta MA , van der Peet DL , Heisterkamp J , Nieuwenhuijzen GAP , et al. Distribution of lymph node metastases in esophageal carcinoma [TIGER study]: study protocol of a multinational observational study. BMC Cancer. 2019;19(1):662. 31272485 10.1186/s12885-019-5761-7 PMC6610993

[62] Shimizu M , Zaninotto G , Nagata K , Graham DY , Lauwers GY . Esophageal squamous cell carcinoma with special reference to its early stage. Best Pract Res Clin Gastroenterol. 2013;27(2):171–86. 23809239 10.1016/j.bpg.2013.03.010

[63] Udagawa H . Sentinel node concept in esophageal surgery: an elegant strategy. Ann Thorac Cardiovasc Surg. 2005;11(1):1–3. 15788960

[64] Takeuchi H , Kitagawa Y . Sentinel node navigation surgery in esophageal cancer. Ann Gastroenterol Surg. 2019;3(1):7–13. 30697605 10.1002/ags3.12206 PMC6345658

[65] Nakamura K , Kato K , Igaki H , Ito Y , Mizusawa J , Ando N , et al. Three‐arm phase III trial comparing cisplatin plus 5‐FU (CF) versus docetaxel, cisplatin plus 5‐FU (DCF) versus radiotherapy with CF (CF‐RT) as preoperative therapy for locally advanced esophageal cancer (JCOG1109, NExT study). Jpn J Clin Oncol. 2013;43(7):752–5. 23625063 10.1093/jjco/hyt061

